# Confirmation of genotypic effects for the bovine *APM1* gene on marbling in Hanwoo cattle

**DOI:** 10.1186/s40781-016-0096-2

**Published:** 2016-04-06

**Authors:** Anam Kwon, Krishnamoorthy Srikanth, Eunjin Lee, Seonkwan Kim, Hoyoung Chung

**Affiliations:** Animal Genomics and Bioinformatics Division, National Institute of Animal Science, Wanju, Jeonbuk, 565-851 Republic of Korea

**Keywords:** Marbling, *APM1*, SNP, Genetic association, Hanwoo

## Abstract

**Background:**

Our previous study had identified the SNP (g.81966377T > C) and indel (g.81966364D > I) located in the promoter of *APM1* to have a significant effect on marbling in Hanwoo. *APM1* encodes an adipocytokine called adiponectin, which plays a significant role in lipogenesis. The aim of this study was to verify and validate the effect of the SNP and indel on marbling and other carcass traits in a large, representative, countrywide population of Hanwoo cattle. The carcass traits measured were marbling (MAR), backfat thickness (BFT), loin eye area (LEA), and carcass weight (CAW).

**Results:**

Primers were designed to amplify 346 bp of the genomic segment that contained the targeted SNP (g.81966377) and the indel (g.81966364). After data curation, the genotypes of 8,378 individuals identified using direct sequencing analysis estimated frequencies for C (0.686) and T (0.314) respectively showing genotype frequencies for CC (0.470), CT (0.430) and TT (0.098). The genotypes were significantly associated with MAR, BFT and LEA. The indel had significant effect on marbling (*P* < .0001) with strong additive genetic effects. The allele frequencies was estimated at (DEL, 0.864) and insertion (INS, 0.136) presenting genotypes of D/D (75.63 %), D/I (21.44 %), and I/I (2.92 %). Significant departure from Hardy-Weinberg equilibrium was not detected for both the SNP and the indel.

**Conclusion:**

The SNP genotypes showed significant association with MAR, BFT and LEA with strong additive genetic effects, while the indel was significantly associated with MAR. The results confirmed that the variants can be used as a genetic marker for improving marbling in Hanwoo.

## Background

*APM1* (Adipose most abundant gene transcript 1) gene codes an adipocytokine called adiponectin, and is secreted from the white adipose tissues [1, 2]. It is involved in lipogenesis, glucose production, insulin sensitivity, inflammatory response and blood circulation [3–5]. The gene is located on the bovine chromosome 1, is 13,256 bp in length and is composed of four exons, and the *APM1* protein is 247 amino acids long [6]. *APM1* was identified to be located nearby the QTL affecting marbling, loin eye area and backfat thickness on BTA1 in Angus [2]. Marker assisted selection has evolved as an important tool in selective breeding for developing high quality beef cattle.

Hanwoo which was a draught animal was selectively bred as beef cattle, with a strong emphasis on meat yield and quality traits [7]. Beef quality in Korea is graded based on intramuscular fat or marbling (MAR), meat color (MCO), fat color (FCO), maturity (MAT), texture (TXT), carcass weight (CAW), back fat thickness (BFT) and loin eye area (LEA). The quality of hanwoo beef in the commercial beef market is directly influenced by the abundance of marbling. Studying the association of polymorphisms in genes involved in physiological or biological process with a quantitative trait like carcass traits can be an effective approach for developing markers for selective breeding [8]. *APM1* has been proposed as the most probable gene for obesity and also was found to have strong linkage with body mass index in humans [9]. Genome wide association studies (GWAS) showed that - SNPs in *APM1* was associated with waist circumference [10] and with bone metabolism in humans [11]. Studies have reported significant effects of SNPs in the promoter of *APM1* on beef carcass traits [2, 6, 12]. These results show that *APM1* is associated with fat metabolism and body mass, and suggests that they may have a strong effect on carcass traits in cattle especially on MAR.

Therefore SNPs identified to have a significant association with carcass traits could be used in the national animal breeding program [13]. Our previous reports found genetic effects of SNPs located in the *APM1* gene on carcass traits in Hanwoo [6]. In particular, the SNP g.81966377T > C revealed a significant association with carcass traits. However in order to use this SNP as a standardized molecular marker its genetic effects has to be verified on a randomly sampled population involving a significant number of samples.

The aim of this study was to verify the genetic effect of the SNP g.81966377T > C and an indel (g.81966364D > I) located in the promoter of *AMP1* on carcass traits in a nationwide randomly sampled Hanwoo cattle.

## Methods

### Animal and carcass traits

The experimental procedures were approved by the ethics and welfare committee of the National Institute of Animal Science (NIAS), Korea. In all, 10,400 muscle tissues of Hanwoo cattle were collected from nine packing facilities of the Korean Animal Products Evaluation (KAPE) [official meat quality grading agency] facilities located throughout Korea, thereby avoiding any regional bias, and so that the sample is representative of the whole country. The meat quality was graded by KAPE based on a national - grading system (http://www.ekape.or.kr/view/eng/system/beef.asp). The samples were collected from the *longissimus thoracis* muscle between the 12^th^ and 13^th^ rib and stored immediately at -70 °C until use. The samples were collected over a period of 3 years from 2013 to 2015. The data collected were (MAR, ranging from 1 (for poor) to 9 (highest quality)), loin eye area (LEA, cm^2^) and backfat thickness (BFT, cm). The average slaughter age of the animals was 31.11 months (male) and 52.54 months (female). The carcass traits of animals used in this study are summarized in Table 1.

**Table 1:** Descriptive summaries for the measurements of carcass traits for Hanwoo cattle

	MAR (1 - 9)	BFT (mm)	LEA (cm^2^)	CAW (kg)	AGE (month)
Male	5.60 ± 1.95	12.64 ± 5.32	90.77 ± 10.86	428.10 ± 50.03	31.11 ± 3.56
Female	4.11 ± 1.80	11.90 ± 5.20	80.64 ± 11.27	336.03 ± 48.69	52.54 ± 22.75

*MAR* Marbling score (1, low to 9, high), *BFT* Backfat thickness, *LEA* Loin eye area, *CAW* Carcass weight

### Genomic DNA isolation

Approximately 1 g of the collected tissue was used for DNA isolation using a commercial kit (Wizard DNA extraction kit, Promega) according to the manufactures guidelines. The integrity of the DNA was checked by agarose gel electrophoresis and a NanoDrop 1000 spectrophotometer (Thermo Scientific, USA). The genomic DNAs were then stored at -70 °C until further use.

### PCR amplification

The bovine *APM1* gene sequence (GenBank: JQ7755868) was retrieved from the Genbank database to design PCR primers using DNA select program of the DNAstar package (Version 6.0). The forward and reverse sequences are CAGCTCGGTACTCATGGGGACAAG and GTGGGAGCTGATGGTGGTAACTGG, respectively. The primers were designed to amplify a 346 bp of the *APM1* promoter region which included the targeted SNP (g.81966377T > C) and indel (g.81966364D > I) that was previously identified [2, 6, 8, 12].

The PCR reaction included 50 ng genomic DNA, 1 x reaction buffer, 2.5 mM dNTP, 10 pmoles of each primers and 1 unit *Taq* DNA polymerase (Genetbio, Korea) in a 20 μl reaction. The PCR condition was as follows; initial-denaturation at 95 °C for 5 min, followed by 35 cycles of denaturation at 95 °C for 45 s, annealing at 57 °C for 30 s and Extension at 72 °C for 1 min and a final extension at 72 °C for 5 mins on a thermal cycler (Veriti®96-well, Applied Biosystems, USA). The PCR product was visualized on an agarose gel stained with a fluorescent dye (Morning bio, Korea) under UV light.

### Genotyping

The remaining PCR products were purified using a PCR purification kit (Nucleogen, Korea) for sequencing analysis with reverse primer. The sequencing reactions were carried out in 10 μl reaction containing 1 μl of purified PCR product, 2 μl of 5 x sequencing buffer, 1.6 pico moles of reverse primer and 0.5 ul Bigdye®Teminator (Applied Biosystem, USA). The sequencing PCR were carried out for 35 cycles of 94 °C for 10 s, 57 °C for 10 s, and 60 °C for 3.5 min. The amplification product was precipitated by isopropanol-ethanol precipitation and reconstituted in 10 μl of formamide. After denaturation at 95 °C for 5 min the sequences were analyzed with ABI3730 XL Genetic Analyzer (Applied Biosystems, USA) at NIAS. The resulting sequences were aligned using SeqMan program of the DNAstar Package (Version 6.0) and the genotypes were determined according to the sequence chromatogram.

### Data analysis

After curating the data for missing phenotypes and genotypes, 8,378 samples were used for the statistical analysis.

The statistical analyses were performed using the Statistical Analysis System [14]. Analysis of Variance (ANOVA) based on general linear model (GLM) was performed to check the genotype effects on carcass traits. The statistical model used was as follows,

$$ \mathrm{Y} = \upmu + \mathrm{G} + \mathrm{S} + \mathrm{bA} + \mathrm{e}, $$

Where;

Y: = observed for the target trait
μ: = overall mean of the target trait
G: = genotype effect
S: = sex effect
b: = the regression coefficient for age
A: = age (covariate)
e: = random error.

Least squares means were compared using Fisher’s least significant difference test with a comparison error rate of 0.005. Additive genetic effects were estimated by the difference between estimates for the two homozygous genotypes and the dominance deviation was estimated by the difference between the solution for the heterozygous genotype and the average of the solutions for the two homozygous.

## Results

### Descriptive summary of carcass traits

The carcass traits of animals used in the analysis are summarized in Table 1. The traits reported are MAR, BFT, CAW, LEA and Age.

### Genotype analysis

**Fig. 1:**
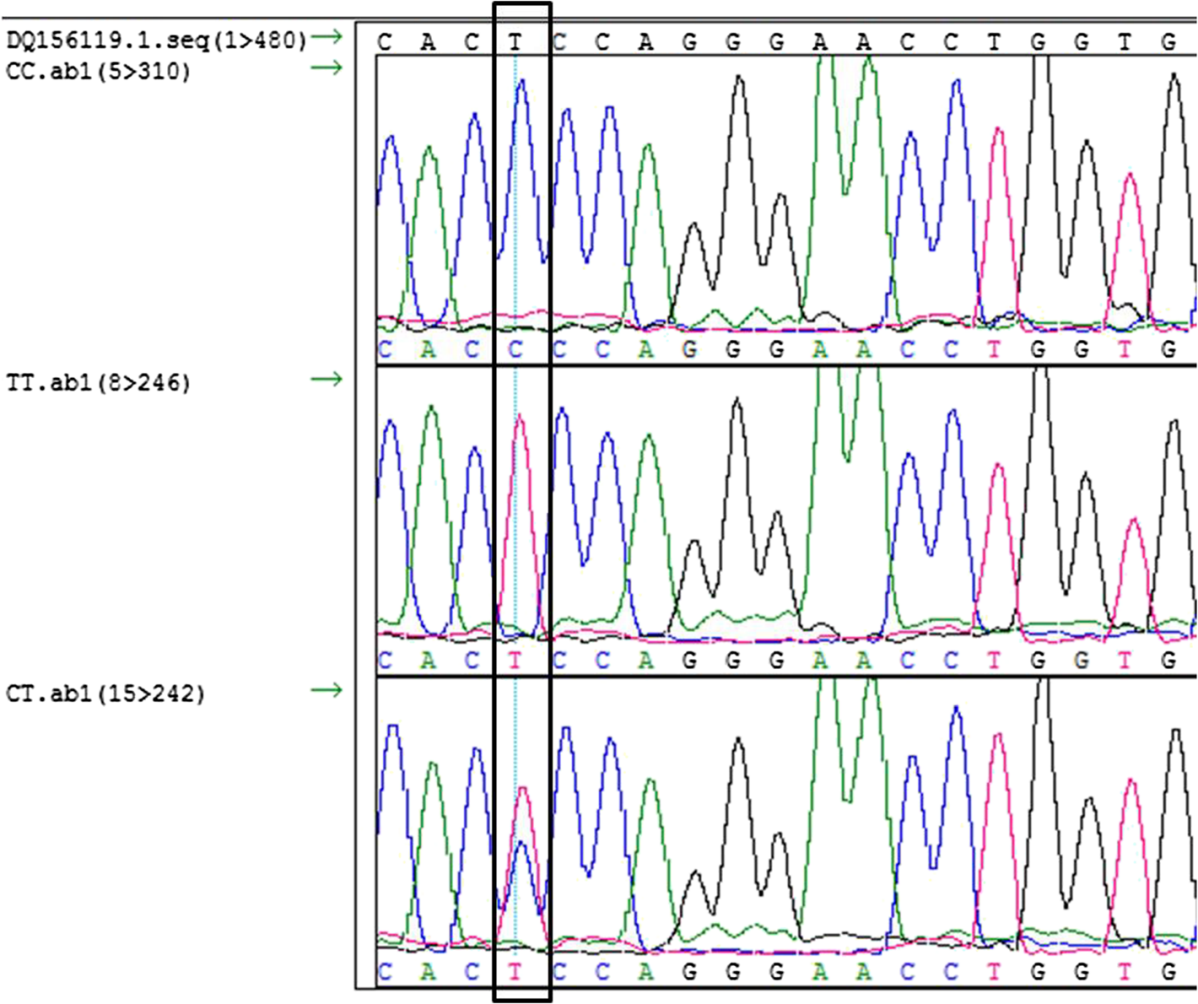
Chromatogram showing three genotypes of g.81966377T > C in Hanwoo *APM1* gene

**Fig. 2:**
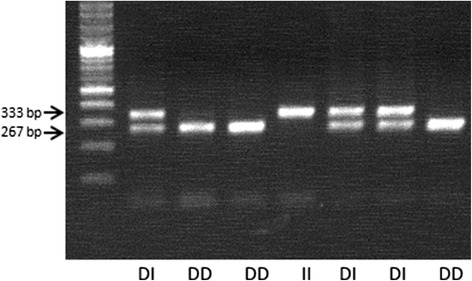
Agarose gel electrophoresis image showing the presence and absence of the 67 bp indel. Amplification produced 267 bp (Deletion) and 333 bp (Insertion)

The sequences were aligned with a reference sequence downloaded from NCBI (DQ156119.1) and the nucleotide substitution at 81966377 (T > C) and the indel (g.81966364D > I) was verified (Figs. 1 and 2). The genotype frequencies for CC, CT and TT were 0.470, 0.430 and 0.098 respectively, while for the indel it was D/D (75.63 %),D/I (21.44 %), and I/I (2.92 %). The allele frequency for the SNP were estimated to be (C, 0.530) and (T, 0.471) and for the indel it was, deletion (DEL, 0.864) and insertion (INS, 0.136). No significant departure from Hardy-Weinberg equilibrium (HWE) was detected. The SNP is listed as an upstream gene variant at a distance of 181 bp from the transcription start site.

### Association analysis

As shown in Table 2 the results showed significant associations of g.81966377T > C with MAR, BFT (*P* < 0.001) and LEA (*P* < 0.05). Animals with genotype CC (4.992) had significant effects on MAR than CT (4.942) and TT (4.693) with a significant additive genetic effect. Animals with genotypes TT (12.55, 86.563) and CT (12.335, 86.325) had significant effects on BFT, LEA than CC (11.430, 85.512) with significant additive effects. The SNP was not significantly associated with CAW, though the genotype TT had the highest effect on CAW followed by genotypes CT and CC. The genotypes of the indel was strongly associated with MAR (*P* < 0.0001) with significant additive effect, while no significant association with BFT, LEA and CAW was detected (Table 2).

**Table 2:** Least squares means and standard errors of marbling, backfat thickness, loineye area, and carcass weight traits according to the genotypes of *APM1* genes in Hanwoo

Trait	g.81966377T > C			*P*	Effect	
	CC (N = 3943)	CT (N = 3610)	TT (N =825)		Additive	Dominance
MAR (1-9)	4.992 ± 0.03^a^	4.942 ± 0.03^b^	4.693 ± 0.04^c^	<0.0001	0.298 ± 0.06***	0.198 ± 0.08
BFT (mm)	11.43 ± 0.13^c^	12.335 ± 0.08^b^	12.558 ± 0.10^a^	<0.0001	1.127 ± 0.16***	0.681 ± 0.23
LEA (cm^2^)	85.512 ± 0.28^b^	86.325 ± 0.22^a^	86.563 ± 0.18^a^	0.0083	0.812 ± 0.36*	1.287 ± 0.51
CAW (kg)	390.646 ± 1.38^c^	392.985 ± 0.87^b^	393.322 ± 1.22^a^	0.2677	2.675 ± 1.75	2.002 ± 2.48
Trait	g.81966364D > I			*P*	Effect	
	D/D (N = 6336)	D/I (N = 1790)	I/I (N = 252)		Additive	Dominance
MAR (1-9)	4.544 ± 0.02a	4.82 ± 0.5^b^	4.885 ± 0.13^b^	<.0001	−0.341 ± 0.14**	0.174 ± 0.14
BFT (mm)	11.815 ± 0.07	11.992 ± 0.13	12.409 ± 0.37	0.179	−0.594 ± 0.37	−0.241 ± 0.46
LEA (cm^2^)	84.388 ± 0.15	84.729 ± 0.29	85.518 ± 0.8	0.259	−1.129 ± 0.81	−0.448 ± 1.01
CAW (kg)	377.349 ± 0.67	377.537 ± 1.27	377.809 ± 3.43	0.984	−0.46 ± 3.5	−0.085 ± 4.32

The carcass traits of Hanwoo cattle were based on a grading system (http://www.ekape.or.kr/view/eng/system/beef.asp) from KAPE (Korean Animal Products Evaluation)

*MAR* marbling score, *BFT* backfat thickness, *LEA* loineye area, *CAW* carcass weight

*P < 0.05, **P < 0.01, and ***P < 0.001

^a,b^ and ^c^ Different letters denote statistically significant differences between genotypes

## Discussion

There has been huge demand for high quality beef from 1970s with increased marbling, taste, and texture [12]. Effective grading mechanisms were put in place by 1994 in order to have an effective measure for improvements achieved. By 2010 significant improvements were achieved, with trait measurements of 407 kg CAW, 13 mm BFT, 86 cm^2^ LEAS and a MAR score of 5.0. In 2013 future livestock improvement goal was announced with the target of reaching a MAR score of 6.5, 90 cm^2^ LEA, 13 mm BFT and 438 kg CAW for hanwoo (http://ebook.mifaff.go.kr/preview/viewer/main.php?site=2&menuno=2&previewno=1381&iframe=0&dlbt=). To achieve this goal, a marker based selection becomes imperative. Though genes having effect on polygenic traits like carcass and meat quality are still unclear, potential candidate have been identified by looking for associations between important traits and some physiological and biochemical processes [8, 15].

*APM1*, which is involved in lipid synthesis, fatty acid oxidation, energy homeostasis, insulin sensitivity and glucose utilization [16] encodes an adiponectin, and is mapped to BTA 1 near the QTL for LEA, BFT and MAR [2]. Thus, *APM1* could be a functional gene that is valuable for meat quality traits especially for fat related traits. Though the direct genetic effect of the studied SNP on the gene is still to be elucidated, variations in the promoter region could directly influence its expression thereby influencing meat quality and quantity traits. Several studies have recorded variations in the bovine *APM1* promoter region [2, 6, 8, 17].

Our previous analysis reported the genetic effect of g.81966377T > C and a 67 bp insertion (g.81966364D > I) in the promoter of *APM1* on meat quality [6] showing significant association with MAR, BFT, LEA and CAW. Thus the findings suggested that the indentified SNP and indel could be used for selection program [13]. However in order for using this SNP as a standardized genetic marker the effect of this variant on carcass traits has to be verified and validated on a large sample set that is representative of the entire Hanwoo population. Unlike our previous study which had used samples from a specific region, in this study samples were collected from KAPE located throughout the country, in all 8,378 samples were genotyped and their association with meat quality traits were analyzed. Significant additive effects were identified for MAR, BFT and LEA. Unlike the previous study the genotypes were not found to have significant association with CAW, though the genotype effect followed a TT < CT < CC which was the same as the previous study, some minor differences in the allele frequency and genotype frequencies from previous work were also identified [6, 8] but these could be attributed to the difference in the population. As for the indel, like the previous study the indel had significant effect only for MAR. Unlike found in the previous study, the sizes of the effect of the SNP and the indel on traits were comparatively smaller, this could be due to the larger sample size or due to the different population used in this study. However the effects were significant, and since they have been validated in a large sample size, they can be used as a marker for marker assisted selection programs. In addition, previous studies have reported strong association of variation in the promoter of *APM1* with growth, LEA and BFT Angus [2] and Chinese cattle breeds [12]. According to the studies *APM1* has been confirmed to regulate bone development, yield grade and weight traits [10, 11, 18, 19]. Our results are in agreement to these previous studies with the studied SNP (g.81966377T > C) showing significant effects on BFT and LEA in Hanwoo. The genotypes of the SNP and indel can be leveraged for selecting animals with favorable traits in Hanwoo industry. Since the effect has been validated on an unbiased randomly selected Hanwoo cattle population from the entire country, this SNP and indel is fit for being used as a robust genetic marker.

## Conclusion

The genetic effect of the SNP (g.81966377T > C) and indel (g.81966364D > I) located in the promoter of *APM1* was verified in 8,378 animals. The genotypes were found to have significant association on MAR, BFT and LEA with strong additive effects, while the indel was found to have a significant association with MAR, paving the way for their use as a robus marker for selective breeding.
